# Changing cluster composition in cluster randomised controlled trials: design and analysis considerations

**DOI:** 10.1186/1745-6215-15-184

**Published:** 2014-05-24

**Authors:** Neil Corrigan, Michael J G Bankart, Laura J Gray, Karen L Smith

**Affiliations:** 1 Department of Health Sciences, University of Leicester, 22-28 Princess Road West, Leicester LE1 6TP, UK; 2 Clinical Trials Research Unit, University of Leeds, Leeds LS2 9JT, UK; 3 Health Services Research Unit, Keele University, Keele ST5 5BG, UK

**Keywords:** Cluster merging, Cluster randomised trials, Loss to follow-up, Primary care, Sample size, Variability in cluster size

## Abstract

**Background:**

There are many methodological challenges in the conduct and analysis of cluster randomised controlled trials, but one that has received little attention is that of post-randomisation changes to cluster composition. To illustrate this, we focus on the issue of cluster merging, considering the impact on the design, analysis and interpretation of trial outcomes.

**Methods:**

We explored the effects of merging clusters on study power using standard methods of power calculation. We assessed the potential impacts on study findings of both homogeneous cluster merges (involving clusters randomised to the same arm of a trial) and heterogeneous merges (involving clusters randomised to different arms of a trial) by simulation. To determine the impact on bias and precision of treatment effect estimates, we applied standard methods of analysis to different populations under analysis.

**Results:**

Cluster merging produced a systematic reduction in study power. This effect depended on the number of merges and was most pronounced when variability in cluster size was at its greatest. Simulations demonstrate that the impact on analysis was minimal when cluster merges were homogeneous, with impact on study power being balanced by a change in observed intracluster correlation coefficient (ICC). We found a decrease in study power when cluster merges were heterogeneous, and the estimate of treatment effect was attenuated.

**Conclusions:**

Examples of cluster merges found in previously published reports of cluster randomised trials were typically homogeneous rather than heterogeneous. Simulations demonstrated that trial findings in such cases would be unbiased. However, simulations also showed that any heterogeneous cluster merges would introduce bias that would be hard to quantify, as well as having negative impacts on the precision of estimates obtained. Further methodological development is warranted to better determine how to analyse such trials appropriately. Interim recommendations include avoidance of cluster merges where possible, discontinuation of clusters following heterogeneous merges, allowance for potential loss of clusters and additional variability in cluster size in the original sample size calculation, and use of appropriate ICC estimates that reflect cluster size.

## Background

Cluster randomised controlled trials (RCTs), in which groups of individuals rather than the individuals themselves are randomised, are conducted for a variety of reasons. The cluster design is often used when an intervention can be administered only to a group, such as a service-wide change or a public health campaign; when there is a risk that an intervention will affect participants in the nonintervention arm; or for reasons of cost or convenience. Such RCTs have a number of methodological challenges in their design, conduct and analysis, discussions of which can be found in a number of texts [1,2]. One issue that has received little attention is the consequence of changes to the composition of clusters after randomisation, including the merging or fragmentation of clusters. Cluster RCTs are relatively common in general practice settings, where general practitioners (GPs) or general practices, rather than individual patients, are the chosen unit of randomisation. Unfortunately, organisational changes are not uncommon in primary care, with some practices merging and others splitting. The number and size of GP practices in the United Kingdom have changed over time, with a reduction by 28% in the number of single-handed GP practices between 2004 and 2009 and a 19% increase in the total number of GPs. There was a 9% decrease in the number of GP practices between 1997 and 2007 [3], however, and organisational changes to meet the challenges of patient care have been actively encouraged [4].

In this article, we focus on the implications of merging clusters for the design and analysis of cluster RCTs. We chose to focus on this effect in cluster RCTs carried out within primary care, because the reduction in the number of GP practices in recent years could result in greater potential for merges to occur in this setting than in other areas where cluster RCTs are frequently used, such as schools, communities, factories and hospitals.

There are few incidences of cluster merging reported in the literature. Using a MEDLINE search (with search terms ‘Trial’ AND ‘primary care’ AND ‘cluster’), we identified reports of completed cluster RCTs in primary care published between 2004 and June 2012, with the start date chosen because 2004 was the year of publication of the Consolidated Standards of Reporting Trials (CONSORT) extension for cluster RCTs [5], which require descriptions of the flow of participants and clusters. We identified 451 potentially useful references in the search.

After assessing the publication texts, we identified 211 reports of cluster RCTs in primary care. From among these, we found only one in which the authors explicitly reported a merge of clusters [6]. Foy *et al.* conducted two parallel cluster RCTs and reported that a practice merge brought together practices that were in the same arm of one RCT and different arms of another. It is not clear whether or how cluster merging was dealt with in their analysis.

To assess the extent of unreported instances of merging clusters in their RCTs, we contacted authors of papers published between 2010 and the present. From among the 67 authors contacted, 27 replied (response rate = 40.3%). Only one of the respondents had experienced a cluster merge in two practices originally randomised to the same trial arm. In the analysis, these two practices were treated as one [7].

Although the number of reported and/or acknowledged incidence of cluster merging is low, it is not obvious how RCT conduct and analysis should be handled when clusters do merge. We suggest that there are a number of simple options available: (1) discontinue recruitment to affected clusters, (2) analyse clusters separately as randomised or (3) analyse the clusters as a new merged cluster. The extent to which merging of clusters might create difficulties is likely to depend on the nature of the cluster merges, the design of the cluster RCT, the arm of the RCT to which the clusters were originally randomised and the timing of the merge. For example, if two primary care practices merge on a purely administrative level, with access to health-care professionals and patient care unaffected, it seems reasonable to continue as if such clusters had not merged and to analyse them as two separate clusters. Other cases may not be so clear-cut, particularly if patient care is reorganised following merging of clusters, resulting in the potential for contamination. In such cases, careful consideration of the design will be needed with regard to the following issues: how recruitment is conducted (identification and enrolment prior to randomisation or recruitment of individuals postrandomisation), cohort or cross-sectional design and the nature of the intervention (for example, at the level of practice/clinician or patient). In most circumstances, it is unlikely that merged clusters will be analysed as one cluster if the clusters were originally randomised to different arms of a RCT, but it might be considered acceptable in a cross-sectional design, in which different patients are included at each measurement time point. The status of participants at the time of the cluster merge (for example, the number who have already completed treatment, the number part way through treatment and the number in follow-up) may also have a bearing on the decision.

In the remainder of this article, we explore statistical issues related to changing cluster composition. Methods and results are described for continuous outcome measures, although similar principles apply to binary outcome measures.

## Methods

### Impact on study design

The aim of any RCT is to obtain an unbiased estimate of the treatment effect with sufficient precision to enable inferences to be made. In order to accomplish this goal, careful consideration of sufficient sample size is required.

The most common approach to calculating the sample size for a cluster RCT involves increasing the number of participants required for an individually randomised trial by an inflation factor called the *design effect*. Details of the sample size calculation can be found elsewhere [8], but we describe them briefly for a continuous outcome measure.

In the following formulae, the effect to be detected is denoted by δ, type I error by α and type II error by β. Calculations are presented for a two-arm RCT, and it is assumed that the continuous outcome *y* follows a normal distribution, $y\sim N\left(\mu_{i},\sigma_{i}^{2}\right)$, in each population *i* (*i* = 1, 2). If samples of size *n*_*i*_ are collected from population *i* (*i* = 1, 2), then the total sample size required is *n* = *n*_1_ + *n*_2_ = (1 + λ)*n*_1_, where λ is the ratio of clusters allocated to each arm of the RCT, σ^2^ is the pooled variance and ξ_*υ*_ denotes the value that satisfies *P*(*Z* > ξ_*υ*_) = υ for *Z* ~ *N*(0, 1), and is given by

(1) $$n=\frac{{\left(1+\lambda \right)}^{2}{\left(\xi_{a/2}+\xi_{\beta }\right)}^{2}\sigma^{2}}{\lambda \delta^{2}}.$$

Withdrawal rates can also be factored into the calculation, so that if the proportion of participants expected to drop out is *w*, the required sample size becomes

(2) $$n=\frac{{\left(1+\lambda \right)}^{2}{\left(\xi_{\alpha /2}+\xi_{\beta }\right)}^{2}\sigma^{2}}{\left(1-w\right)\lambda \delta^{2}}.$$

For a cluster RCT with equal cluster sizes of *m*, the design effect is given by 1 + (*m* − 1)ρ, where ρ is the intracluster correlation coefficient (ICC) and is calculated as $\rho =\frac{\sigma_{b}^{2}}{\sigma_{b}^{2}+\sigma_{w}^{2}}$, where $\sigma_{b}^{2}$ is the between-cluster variance, $\sigma_{w}^{2}$ is the within-cluster variance and therefore the total variance is given by $\sigma^{2}=\sigma_{b}^{2}+\sigma_{w}^{2}$. The ICC is the proportion of the total variance that is due to between-cluster variability. The sample size required for a cluster RCT becomes

$$n=\frac{{\left(1+\lambda \right)}^{2}{\left(\xi_{\alpha /2}+\xi_{\beta }\right)}^{2}\sigma^{2}}{\lambda \delta^{2}}\left[1+\left(m-1\right)\rho \right].$$

Accounting for within-cluster attrition, *w*, assuming that attrition occurs uniformly across clusters, the calculation becomes

(3) $$n=\frac{{\left(1+\lambda \right)}^{2}{\left(\xi_{a/2}+\xi_{\beta }\right)}^{2}\sigma^{2}}{\left(1-w\right)\lambda \delta^{2}}\left[1+\left(m\left(1-w\right)-1\right)\rho \right].$$

Equivalently, when cluster sizes are fixed, the total number of clusters required is *c*, where

(4) $$c=\frac{{\left(1+\lambda \right)}^{2}{\left(\xi_{\alpha /2}+\xi_{\beta }\right)}^{2}\sigma^{2}}{m\left(1-w\right)\lambda \delta^{2}}\left[1+\left(m\left(1-w\right)-1\right)\rho \right],$$

and power can be calculated as

(5) $$1-\beta =\Phi \left(\sqrt{\frac{m\left(1-w\right)\mathrm{c\lambda}\delta^{2}}{{\left(1+\lambda \right)}^{2}\sigma^{2}\left[1+\left(m\left(1-w\right)-1\right)\rho \right]}}-\xi_{\alpha /2}\right),$$

where Φ is the cumulative distribution function for the standard normal distribution.

Letting

(6) $$\gamma \left(m,c,\lambda ,w,\delta ,\rho \right)=\frac{m\left(1-w\right)\mathrm{c\lambda}\delta^{2}}{{\left(1+\lambda \right)}^{2}\sigma^{2}\left[1+\left(m\left(1-w\right)-1\right)\rho \right]}$$

it can be seen that the study power increases monotonically as γ increases. Thus the impact of each parameter on study power can be examined. The parameters that will be changed by merging clusters are *m*, which for post–cluster merges will be the average cluster size (rather than fixed); *c*, the number of clusters; and λ, the allocation ratio of clusters.

Upon inspection (equation (5)), a simple monotonic relationship between *c* and power is apparent, such that (1 − β) → 1 as *c* → ∞ and (1 − β) → Φ(−ξ_α/2_) as *c* → 0.

It is known that, at larger values of *m*, the benefit gained from further increasing the average cluster size becomes less as the study power plateaus [9]. This has important consequences for study power if clusters merge and the average cluster size increases.

The relationship between study power and the ratio of clusters allocated to each arm of a RCT is nonmonotonic, with optimum power at λ = 1, and decreases in power occurring as the value of λ deviates further from 1. Holding other parameters constant in equation (6), $\gamma \left(\lambda \right)\propto \frac{\lambda }{{\left(1+\lambda \right)}^{2}}$ and $\gamma \prime \left(\lambda \right)\propto \frac{1-\lambda }{{\left(1+\lambda \right)}^{3}}$, which is positive for λ ∈ [0, 1), negative for λ ∈ (1, ∞) and equal to 0 when λ = 1, indicating that γ(λ), and therefore the study power, reaches a maximum point at λ = 1.

Clearly, if a cluster RCT starts with equal-sized clusters and two or more clusters merge, cluster sizes are no longer equal. Variability in cluster size has a detrimental effect on study power, as shown by Kerry and Bland [10]. If cluster sizes follow an underlying distribution with mean *m*_*c*_ and standard deviation σ_*c*_, and if treatment groups have equal numbers of clusters (λ = 1), then, as has been shown by a number of authors [11-14], the design effect becomes

(7) $$1+\left[\left(\frac{\sigma_{c}^{2}}{m_{c}^{2}}+1\right)m_{c}-1\right]\rho ,$$

and it is clear that the design effect increases as the variation in cluster sizes increases. As clusters merge, cluster size variability increases and the design effect also increases. This implies that, without an increase in sample size, study power will decrease following cluster merges.

Consider a scenario in which all clusters are of equal size *m* and treatment groups receive equal allocations of the number of clusters. Let the number of clusters be *c* ∈ 2ℕ and *n*^(*i*)^ denote the size of cluster *i* (*i* = 1, …, *c*). The standard deviation of cluster size, σ_*c*_, is therefore 0.

Suppose that $k\in \left[0,\frac{c}{2}\right]$ pairs of clusters merge, leaving *c* − *k* clusters in total with $n^{\left(j_{1}\right)}=\cdots =n^{\left(j_{k}\right)}=2m$ for *j*_1_, …, *j*_*k*_ ∈ {1, …, *c* − *k*}, and *n*^(*i*)^ = *m* for *i* ∈ {1, …, *c* − *k*}∖{*j*_1_, … *j*_*k*_}. For notational convenience, let *S* denote the set {1, …, *c* − *k*}∖{*j*_1_, …*j*_*k*_}.

The average cluster size following the merge is then

(8) $$\begin{array}{ll} \tilde{m} & =\frac{1}{c-k}\left(\sum_{i\in S}n^{\left(i\right)}+\sum_{i=1}^{k}n^{\left(j_{i}\right)}\right)\\ & =\frac{1}{c-k}\left(\left(c-2k\right)m+2\mathrm{km}\right)=\frac{c}{c-k}m \end{array}$$

Recall that study power increases monotonically with *c*, and, holding all other terms in equation (6) fixed, we obtain $\gamma =\frac{m\left(1-w\right)c}{1+\left(m\left(1-w\right)-1\right)\rho }\times \mathrm{constant}$. After $k\in \left[0,\frac{c}{2}\right]$ merges, this equation becomes $\tilde{\gamma }=\frac{\tilde{m}\left(1-w\right)\left(c-k\right)}{1+\left(\tilde{m}\left(1-w\right)-1\right)\rho }\times \mathrm{constant}$. Note that the constant terms in the expressions for γ and $\tilde{\gamma }$ are equal, but $\tilde{m}\left(c-k\right)=\frac{c}{c-k}m\left(c-k\right)=\mathrm{mc}$. Therefore, $\tilde{\gamma }=\frac{m\left(1-w\right)c}{1+\left(\tilde{m}\left(1-w\right)-1\right)\rho }\times \mathrm{constant}$, and $\tilde{\gamma }\leq \gamma$, with equality holding only if *k* = 0. Hence, power will be reduced following cluster merges if no additional clusters are recruited.

Let ${\tilde{s}}_{c}^{2}$ denote the sample variation in cluster size postmerge. Then

(9) $$\begin{array}{l} {\tilde{s}}_{c}^{2}=\frac{1}{\left(c-k\right)-1}\sum_{i=1}^{c-k}{\left(n^{\left(i\right)}-\tilde{m}\right)}^{2}\\ =\frac{1}{c-k-1}\left(\sum_{i\in S}{\left(n^{\left(i\right)}-\tilde{m}\right)}^{2}+\sum_{i=1}^{k}{\left(n^{\left(j_{i}\right)}-\tilde{m}\right)}^{2}\right)\\ =\frac{1}{c-k-1}\left(\left(c-2k\right){\left(m-\frac{c}{c-k}m\right)}^{2}+k{\left(2m-\frac{c}{c-k}m\right)}^{2}\right)\\ =\frac{m^{2}k\left(c-2k\right)}{\left(c-k\right)\left(c-k-1\right)} \end{array}.$$

**Figure 1:**
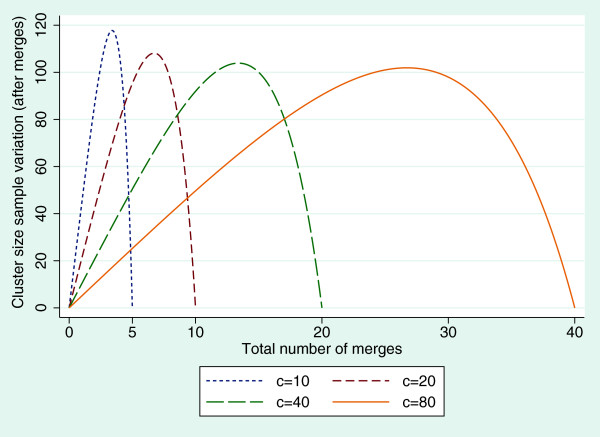
**Relationship between the variability in cluster size and the number of cluster merges.** Graph showing the variability when the number of clusters before the merges take place is *c* = 10, 20, 40 and 80 for fixed cluster size before the merges of *m* = 20.

The variation in cluster size increases as *k* increases from 0, reaching its maximum before decreasing as $k\to \frac{c}{2}$ (see Figure 1).

If the number of cluster merges differs between the treatment groups, then the ratio of the number of clusters λ will also be affected. Let *k*_*i*_ denote the number of merges of cluster pairs in treatment group *i* and *c*_*i*_ denote the number of clusters in treatment group *i* before the merges. Then, after the merges,

$$\tilde{\lambda }=\frac{c_{1}-k_{1}}{c_{2}-k_{2}}=\frac{c-2k_{1}}{c-2k_{2}}.$$

Because optimum power is achieved when λ = 1, if the number of cluster merges is unequal between the treatment groups, the study power will be adversely affected.

These formulae have been used to explore the combined impact of the changes in design parameters graphically.

### Impact on analysis: simulation study

Most of the few reported instances of cluster merges involved clusters within the same treatment arm of a RCT (which we refer to as *homogeneous merging*). In the one instance in which data analysis was reported, the resulting data were analysed with the merged cluster treated as a single cluster. We explored, by simulation, the appropriateness of this pragmatic strategy and considered approaches to analysis when clusters merge that were randomised to different treatment groups (that is, heterogeneous merging).

Cluster RCT data were simulated using the framework of a multilevel model with a simulated two-arm RCT, comprising a control group and an intervention group. Clusters were set to be of equal size with equal allocation of clusters to treatment arms. The outcome for each individual was generated as the sum of three components, $Y_{\mathrm{ij}}=\mu_{\mathrm{ij}}^{\mathrm{trt}}+u_{0j}+\epsilon_{0\mathrm{ij}}$, where $\mu_{\mathrm{ij}}^{\mathrm{trt}}$ was the mean outcome for the treatment group to which patient *i* in cluster *j* was allocated, *u*_0*j*_ was sampled from $N\left(0,\sigma_{b}^{2}\right)$ and represented the cluster-level error for all individuals in that cluster, and *ϵ*_0*ij*_ was sampled from $N\left(0,\sigma_{w}^{2}\right)$ and was used as the individual-level error. Without loss of generality, $\sigma_{b}^{2}$ and $\sigma_{w}^{2}$ were chosen so that their sum was equal to 1. A value of 0.05 was used for the ICC, a commonly used value in designing cluster RCTs in primary care. The total number of clusters was set at 80, and 20 individuals were allocated to each cluster, giving a 5% significance level at 80% power and an effect size of 0.2. True treatment group means were given the values μ_0_ = 0 and μ_1_ = 0.2.

For each scenario, 1,000 simulations were generated and a random intercept model was fitted to the resultant data sets. For model-fitting, we used restricted maximum likelihood to improve estimates of the variance components [15].

We conducted further simulations, keeping the planned power static at 80% but increasing the cluster size with a corresponding reduction in the number of clusters. This resulted in combinations of 60 clusters with 40 individuals per cluster and 48 clusters with 100 individuals per cluster.

#### Homogeneous merges

Homogeneous cluster merges alter cluster size, average cluster size and, potentially, ICC, all of which have an impact on study power.

##### Scenario 1

For each homogeneous merge, two clusters from the same treatment group, which had not already been involved in a merge, were selected at random to become a merged cluster. Individual patient outcomes were left unchanged because it is assumed that treatment is not affected by the merge of clusters. The scenario was simulated for all pairs (*k*_0_, *k*_1_) ∈ *M* × *M*, where *M* = {0, 1, 2, 5, 10, 20} and *k*_0_ and *k*_1_ are the number of merges in the control and intervention groups, respectively.

##### Scenario 2

A further scenario was simulated, to more closely reflect what might happen in practice. In this scenario, half of the individuals were assumed to have completed treatment prior to cluster merge, retaining the old cluster level error term. The remainder were allocated to a new merged cluster with a new cluster level error term applied in generating the outcome.

#### Heterogeneous merges

Two different scenarios were used to simulate heterogeneous cluster merges.

##### Scenario 3

The simulated data sets were adjusted in a similar way as that used for homogeneous merges, with each merge consisting of one cluster from the control arm and one from the intervention arm randomly selected to form a merged cluster. With this scenario, whilst unrealistic in practice and presented here as an extreme illustration, we assumed that patient outcomes are unchanged following a merge and represented a RCT in which all patients completed the intervention prior to a merge.

Three strategies for analysis were explored: (1) merged clusters were allocated to the control arm of the study, (2) merged clusters were allocated to the intervention arm of the study or (3) merged clusters were eliminated from the analysis. It was expected that the first two strategies would lead to bias and that the third, whilst unbiased, would lead to a loss of power.

##### Scenario 4

Rather than assume that all patients completed the intervention prior to the merge, in this scenario, we assumed that only 50% of the patients did so. The treatment group mean component used to simulate outcomes for individuals not completing treatment prior to the merge was adjusted according to treatment group allocation postmerge. As with scenario 3, analysis was based on three strategies: (1) merged clusters were allocated to the control group, (2) merged clusters were allocated to the intervention group or (3) merged clusters were dropped from the analysis.

Additionally, this scenario was simulated both with and without those who did not complete treatment prior to the merge, with individuals analysed according to their original cluster assignment when noncompleters were omitted. This analysis reflected a pragmatic approach of discontinuing clusters following a merge.

As with homogeneous merges, further simulations were conducted with increased cluster size and a reduced number of clusters, keeping the planned study power constant at 80%. All simulations and analyses were conducted using Stata 12 software (StataCorp, College Station, TX, USA). Example code for the simulations is given in Additional file 1.

## Results

### Study design

**Figure 2:**
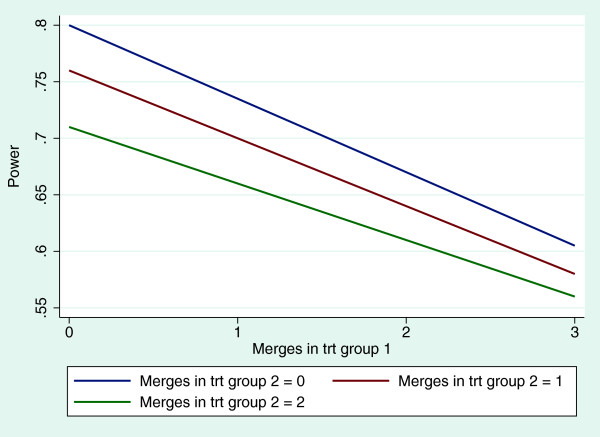
**Relationship between power and number of cluster merges.** Graph shows three cluster merges in treatment (trt) group 1 and two cluster merges in treatment group 2.

Equation (6) allowed exploration of the variables that affect study power when clusters are merged. Figure 2 illustrates this impact when, for example, up to three merges in treatment group 1 and two merges in treatment group 2 occur. We note that the effect is as expected on the basis of equation (6). There is a linear effect on power at each level of merges in each treatment group, and adjustment for potential cluster merges in study design would be straightforward, although the choice of assumptions to be made in practice with regard to potential numbers of cluster merges is more difficult.

### Analysis: simulation study

We analysed the complete simulated data sets without cluster merges. From among the 1,000 simulated data sets, 826 yielded evidence of a significant treatment difference at the 5% level, and parameter estimates were all in agreement with the ‘true’ level.

### Homogeneous cluster merges

#### Scenario 1

Empirical parameter estimates based on equal numbers of cluster merges are given in Table 1. Estimates of the treatment effect β_1_ were unbiased, as expected, with estimates of β_0_ being consistent with the ‘true’ value of 0.

**Table 1:** **Parameter estimates following homogeneous cluster merges: scenario 1**^**a**^

	**Number of cluster merges per treatment group**					
**Empirical estimates**	**0**	**1**	**2**	**5**	**10**	**20**
Intercept, β_0_	0.001 (−0.003, 0.005)	0.000 (−0.003, 0.004)	−0.001 (−0.005, 0.002)	−0.001 (−0.004, 0.002)	0.000 (−0.003, 0.004)	−0.003 (−0.006, 0.001)
Treatment effect, β_1_	0.200 (0.195, 0.205)	0.200 (0.195, 0.205)	0.201 (0.196, 0.206)	0.202 (0.198, 0.207)	0.201 (0.196, 0.206)	0.204 (0.199, 0.209)
$\sigma_{b}^{2}$	0.050 (0.048, 0.051)	0.049 (0.048, 0.051)	0.047 (0.046, 0.049)	0.044 (0.043, 0.045)	0.038 (0.037, 0.040)	0.024 (0.023, 0.025)
$\sigma_{w}^{2}$	0.950 (0.947, 0.952)	0.952 (0.949, 0.954)	0.954 (0.951, 0.957)	0.955 (0.953, 0.958)	0.962 (0.959, 0.965)	0.975 (0.973, 0.978)
Intracluster correlation coefficient	0.050 (0.048, 0.051)	0.049 (0.048, 0.051)	0.047 (0.046, 0.049)	0.044 (0.042, 0.045)	0.038 (0.037, 0.039)	0.024 (0.023, 0.025)
Cluster size variance	0	10.1	20.2	49.7	90.4	0
Empirical power	81.8%	80.0%	82.0%	81.7%	80.9%	83.9%

^a^Patient outcomes are assumed to be unaffected by cluster merge. Total of 80 clusters with 20 patients in each prior to cluster merges. Data are mean (95% confidence interval). $\sigma_{b}^{2}$ is the between-cluster variance and $\sigma_{w}^{2}$ is the within-cluster variance.

**Figure 3:**
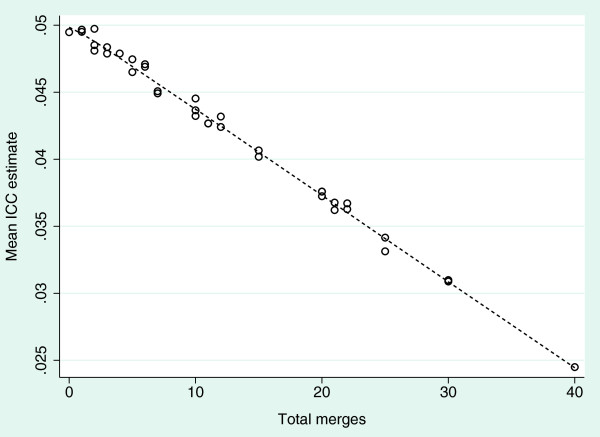
**Mean intracluster correlation coefficient estimate by total number of homogeneous cluster merges.** Graphed data derived from simulations, including all pairs of (*k*_0_,*k*_1_) ∈ *M* × *M* of numbers of merges in each treatment group, with the overlay created using the Lowess (locally weighted scatterplot smoothing) procedure. ICC, Intracluster correlation coefficient.

The variance component estimates have been affected by the cluster merges, with the between cluster variability, $\sigma_{b}^{2}$, decreasing as the number of merges increases. Since the total variation at the individual level is unaffected, the within cluster variability, $\sigma_{w}^{2}$, increased. Consequently the ICC also decreased. Figure 3 shows the relationship between the estimate of ICC and the total number of cluster merges and it appears that the ICC depends on the average cluster size. Although there was a small impact on study power, this is rather less than expected from the results presented earlier, and was explained by the change to ICC.

**Figure 4:**
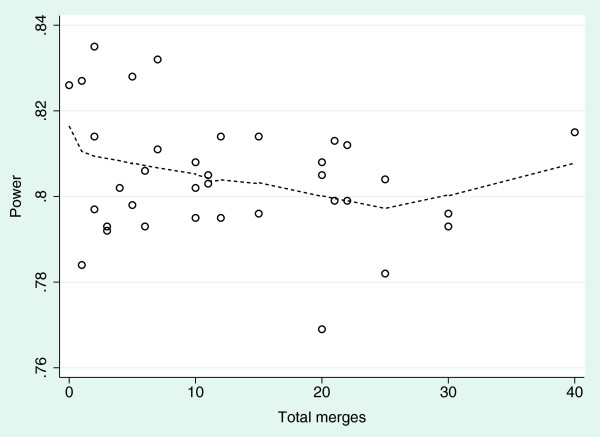
**Observed study power by number of homogeneous cluster merges.** Data including all pairs of (*k*_0_,*k*_1_) ∈ *M* × *M* of numbers of merges in each treatment group, with the overlay created using the Lowess procedure.

Simulations in which unequal numbers of cluster merges occurred in each of the intervention groups gave broadly similar results, albeit with a greater loss of power as the imbalance in cluster allocation to treatments increased. The effect on power is illustrated in Figure 4.

The same patterns in estimates were observed when the number and size of clusters was varied, with very similar results by proportion of clusters merging.

#### Scenario 2

When only 50% of patients are assumed to have completed prior to a merge a similar pattern was observed.

### Heterogeneous cluster merges

#### Scenario 3

When each pair of merges consisted of one cluster from the control arm and one from the intervention arm, assuming patient outcomes are unaffected by the merge, the simulations demonstrated attenuation of the treatment effect if all merged clusters were assigned to one of the treatment groups, with the treatment effect estimate decreasing as the number of merges increased (Table 2). Unsurprisingly, the control group estimate was biased when merged clusters were allocated to the control group. If the resulting merged clusters were dropped from the analysis, the treatment effect estimate was unbiased, as expected, but with a loss of precision.

**Table 2:** **Parameter estimates following heterogeneous cluster merges: scenario 3**^**a**^

	**Number of cluster merges per treatment group**					
**Empirical estimates**	**0**	**1**	**2**	**5**	**10**	**20**
Assigned to control						
Intercept, β_0_	0.001 (−0.003, 0.004)	0.004 (0.000, 0.007)	0.006 (0.002, 0.009)	0.015 (0.012, 0.019)	0.032 (0.029, 0.035)	0.057 (0.054, 0.060)
Treatment effect, β_1_	0.199 (0.195, 0.204)	0.197 (0.192, 0.202)	0.194 (0.189, 0.198)	0.186 (0.181, 0.190)	0.170 (0.165, 0.175)	0.001 (−0.003, 0.004)
$\sigma_{b}^{2}$	0.051 (0.049, 0.052)	0.050 (0.049, 0.051)	0.049 (0.047, 0.0498)	0.048 (0.046, 0.049)	0.046 (0.045, 0.047)	0.039 (0.038, 0.040)
$\sigma_{w}^{2}$	0.951 (0.948, 0.954)	0.951 (0.949, 0.954)	0.953 (0.951, 0.956)	0.953 (0.951, 0.956)	0.958 (0.955, 0.961)	0.969 (0.966, 0.971)
Intracluster correlation coefficient	0.050 (0.048, 0.051)	0.050 (0.049, 0.051)	0.049 (0.047, 0.0494)	0.048 (0.046, 0.049)	0.046 (0.044, 0.047)	0.039 (0.037, 0.040)
Empirical power	82.5%	79.0%	78.8%	74.3%	64.3%	46.9%
Assigned to intervention						
Intercept, β_0_	0.002 (−0.002, 0.005)	−0.002 (−0.006, 0.001)	0.000 (−0.004, 0.003)	0.001 (−0.003, 0.005)	0.000 (−0.004, 0.004)	−0.003 (−0.008, 0.001)
Treatment effect, β_1_	0.201 (0.197, 0.206)	0.198 (0.194, 0.203)	0.192 (0.187, 0.196)	0.185 (0.180, 0.190)	0.166 (0.161, 0.171)	0.146 (0.141, 0.152)
$\sigma_{b}^{2}$	0.050 (0.048, 0.051)	0.050 (0.048, 0.051)	0.049 (0.047, 0.0499)	0.047 (0.046, 0.049)	0.045 (0.043, 0.046)	0.039 (0.038, 0.040)
$\sigma_{w}^{2}$	0.948 (0.946, 0.950)	0.950 (0.947, 0.952)	0.950 (0.948, 0.953)	0.956 (0.953, 0.958)	0.958 (0.956, 0.961)	0.968 (0.965, 0.970)
Intracluster correlation coefficient	0.049 (0.048, 0.051)	0.050 (0.048, 0.051)	0.049 (0.047, 0.0498)	0.047 (0.046, 0.048)	0.045 (0.043, 0.046)	0.039 (0.037, 0.040)
Empirical power	82.7%	80.7%	79.5%	72.9%	64.2%	45.1%
Dropped from analysis						
Intercept, β_0_	0.001 (−0.003,0.004)	−0.002 (−0.006, 0.001)	−0.001 (−0.004, 0.003)	−0.004 (−0.007, −0.001)	−0.001 (−0.005, 0.003)	0.000 (−0.004, 0.005)
Treatment effect, β_1_	0.202 (0.197, 0.207)	0.201 (0.196, 0.206)	0.199 (0.194, 0.204)	0.202 (0.197, 0.207)	0.202 (0.196, 0.207)	0.198 (0.192, 0.205)
$\sigma_{b}^{2}$	0.051 (0.049, 0.052)	0.050 (0.048, 0.051)	0.050 (0.048, 0.051)	0.049 (0.048, 0.051)	0.049 (0.047, 0.051)	0.050 (0.048, 0.051)
$\sigma_{w}^{2}$	0.949 (0.947, 0.952)	0.951 (0.948, 0.953)	0.952 (0.950, 0.955)	0.950 (0.947, 0.953)	0.953 (0.950, 0.956)	0.950 (0.946, 0.953)
Intracluster correlation coefficient	0.051 (0.049, 0.052)	0.050 (0.048, 0.051)	0.050 (0.048, 0.051)	0.049 (0.048, 0.051)	0.049 (0.047, 0.0499)	0.049 (0.048, 0.051)
Empirical power	81.7%	81.0%	78.3%	77.6%	70.2%	53.1%

^a^Patient outcomes are assumed to be unaffected by cluster merge, indicating that all treatments were finished prior to merge. Total of 80 clusters with 20 patients in each cluster prior to cluster merges. Data are mean (95% confidence interval). $\sigma_{b}^{2}$ is the between-cluster variance and $\sigma_{w}^{2}$ is the within-cluster variance.

As with the homogeneous merges, the ICC decreased as the total number of merges increased, but in this scenario the decrease was not sufficient to prevent the severe loss of power caused by the merges.

#### Scenario 4

Under the assumption of 50% of patients completing treatment prior to merges of one cluster from the control arm with one cluster from the intervention arm, the impact of the heterogeneous merges on the treatment effect estimates again was attenuation of the treatment effect, but less extreme than under scenario 3 (Table 3). Variance components were affected as before, and the impact on study power, whilst substantial, was not as severe as it was under scenario 3.

**Table 3:** **Parameter estimates following heterogeneous cluster merges: scenario 4**^**a**^

	**Number of cluster merges per treatment group**					
**Empirical estimates**	**0**	**1**	**2**	**5**	**10**	**20**
Assigned to control						
Intercept, β_0_	0.001 (−0.003, 0.004)	0.003 (0.000, 0.007)	0.003 (0.000, 0.007)	0.007 (0.004, 0.011)	0.017 (0.013, 0.020)	0.035 (0.032, 0.0380)
Treatment effect, β_1_	0.201 (0.196, 0.205)	0.196 (0.191, 0.199)	0.196 (0.191, 0.201)	0.191 (0.186, 0.196)	0.184 (0.179, 0.189)	0.166 (0.159, 0.172)
$\sigma_{b}^{2}$	0.050 (0.048, 0.051)	0.049 (0.048, 0.051)	0.049 (0.047, 0.0496)	0.046 (0.045, 0.047)	0.043 (0.042, 0.044)	0.034 (0.033, 0.036)
$\sigma_{w}^{2}$	0.950 (0.948, 0.952)	0.951 (0.950, 0.953)	0.952 (0.950, 0.954)	0.957 (0.954, 0.959)	0.962 (0.959, 0.964)	0.971 (0.969, 0.974)
Intracluster correlation coefficient	0.050 (0.048, 0.051)	0.049 (0.048, 0.0499)	0.049 (0.047, 0.0494)	0.046 (0.044, 0.047)	0.042 (0.041, 0.044)	0.034 (0.033, 0.035)
Empirical power	81.2%	78.1%	78.3%	76.8%	72.1%	48.2%
Assigned to intervention						
Intercept, β_0_	0.003 (<0.001, 0.007)	0.001 (−0.003, 0.004)	0.002 (−0.002, 0.006)	−0.002 (−0.006, 0.001)	0.001 (−0.004, 0.005)	−0.004 (−0.010, 0.002)
Treatment effect, β_1_	0.198 (0.193, 0.202)	0.195 (0.191, 0.1997)	0.194 (0.189, 0.199)	0.192 (0.187, 0.197)	0.179 (0.174, 0.184)	0.168 (0.162, 0.175)
$\sigma_{b}^{2}$	0.049 (0.048, 0.051)	0.050 (0.048, 0.051)	0.048 (0.047, 0.0493)	0.047 (0.046, 0.049)	0.044 (0.042, 0.045)	0.034 (0.033, 0.035)
$\sigma_{w}^{2}$	0.949 (0.947, 0.951)	0.951 (0.949, 0.953)	0.953 (0.951, 0.955)	0.956 (0.954, 0.958)	0.963 (0.961, 0.965)	0.973 (0.971, 0.976)
Intracluster correlation coefficient	0.049 (0.048, 0.050)	0.049 (0.048, 0.051)	0.048 (0.047, 0.049)	0.047 (0.046, 0.048)	0.043 (0.042, 0.044)	0.033 (0.032, 0.035)
Empirical power	81.0%	79.7%	77.3%	75.8%	67.2%	50.7%
Completers only						
Intercept, β_0_	−0.000 (−0.004, 0.003)	0.004 (0.000, 0.007)	0.001 (−0.003, 0.005)	−0.000 (−0.004, 0.003)	0.001 (−0.002, 0.005)	−0.000 (−0.004, 0.003)
Treatment effect, β_1_	0.200 (0.195, 0.205)	0.195 (0.190, 0.1991)	0.198 (0.193, 0.203)	0.199 (0.194, 0.204)	0.198 (0.193, 0.203)	0.200 (0.194, 0.205)
$\sigma_{b}^{2}$	0.050 (0.049, 0.051)	0.050 (0.048, 0.051)	0.050 (0.049, 0.051)	0.051 (0.049, 0.052)	0.050 (0.049, 0.051)	0.050 (0.048, 0.051)
$\sigma_{w}^{2}$	0.950 (0.948, 0.952)	0.952 (0.950, 0.954)	0.949 (0.947, 0.952)	0.950 (0.947, 0.952)	0.951 (0.948, 0.953)	0.948 (0.946, 0.951)
Intracluster correlation coefficient	0.050 (0.049, 0.051)	0.050 (0.048, 0.051)	0.050 (0.049, 0.051)	0.050 (0.049, 0.052)	0.050 (0.048, 0.051)	0.050 (0.048, 0.051)
Empirical power	82.3%	79.2%	79.6%	79.5%	77.1%	73.3%

^a^In scenario 4, 50% of patient outcomes are assumed to be unaffected by cluster merge, which is akin to 50% completing treatment prior to merge, with the remainder allocated treatment group mean based on postmerge treatment group allocation. Total of 80 clusters with 20 patients in each prior to cluster merges. Data are mean (95% confidence interval). $\sigma_{b}^{2}$ is the between-cluster variance and $\sigma_{w}^{2}$ is the within-cluster variance.

If the analysis is restricted to those completing treatment prior to the cluster merge (labelled “Completers only” in Table 3), then the treatment effect estimates remained unbiased as expected, but the estimates are less precise because of the effective reduction in sample size. The ICC is unaffected by the number of merges, and study power is slightly affected. As with homogeneous merges, the same patterns in estimates were observed when the number and size of clusters were varied, with very similar results by proportion of clusters merging.

## Discussion

We have demonstrated, through established approaches to power calculation, that cluster merges have an adverse impact on study power, assuming that the ICC is unaffected by the change in average cluster size and variability in cluster size. Given the way in which study power may be impacted if clusters merge, we suggest that allowance in this case may need to be made through recruitment of additional clusters rather than just by increasing the size of the clusters, which is the more common approach when allowing for loss to follow-up, although a combination of the two may need to be considered. This issue is closely related to that of variability in cluster size and loss to follow-up of clusters, in effect being a combination of the two. Consequently, the basis of allowance for cluster merges in the design could be through using established, previously published methods such as the one proposed by Taljaard *et al.*[16]. However, given the cost of additional clusters, we suggest that the decision whether to allow for cluster merging will depend on the perceived likelihood of merges in any particular study and will be based on knowledge of the chosen participating sites.

The simulations suggest that homogeneous cluster merges do not affect the treatment effect estimate. In our present analysis, we assumed that the cluster size represents the whole cluster for each cluster, not just a subset of a larger cluster being analysed. Consequently, the anticipated loss in study power was offset by the change in the ICC, such that the impact was much smaller than expected. The linear relationship obtained between the estimate of ICC and the total number of cluster merges indicates that the ICC depends on the average cluster size. This is in keeping with the relationship between ICC and natural cluster size that has been shown previously [17,18], with smaller ICC as the average cluster size increases. This change in ICC would not occur if the size of the cluster represented the number from a larger cluster being analysed, because ICC is related to the natural cluster size rather than the number sampled, and, in such circumstances, we would expect to see a loss in study power following any merges.

The simulations therefore indicate that the pragmatic approach to analysis, treating the new merged cluster as one cluster, if any homogeneous cluster merges occur is reasonable, without causing bias or loss of precision in treatment effect estimate.

The attenuation of the treatment effect estimate following heterogeneous cluster merges is unsurprising, given the change in cluster composition, although we note that the impact is minimal when there are only a few cluster merges. For example, under scenario 3, the clusters resulting from the merge consist of an equal number of individuals from each treatment group, and we might then expect the outcome in these clusters to be (μ_0_ + μ_1_)/2. Following assignment to either treatment group, the treatment effect will be attenuated, either through an increase in mean response in the control group or a decrease in mean response in the intervention group. Consequently, assigning merged clusters to either treatment group in these circumstances will result in biased estimates.

Bias following heterogeneous merges can be avoided by dropping merged clusters from the analysis or by including only those individuals who completed treatment prior to the merge. In practice, this would require that any merged clusters discontinue the RCT.

In a review of 152 cluster RCTs in primary care, Eldridge *et al.* reported an average cluster size of 32 and an interquartile range of 9 to 82 [19]. In our present study, we assessed three fixed cluster sizes—20, 40 and 100—that reflect the cluster sizes in RCTs carried out in primary care. We note that the findings in each scenario were dependent not on cluster size, only on the proportion of clusters merging. We would not expect the impact to be any different with larger cluster sizes.

We have assumed a fixed cluster size, that is, that the number of individuals recruited per cluster is the same across clusters. In some RCTs, this may be unrealistic, such as in situations where an entire GP practice is included. A review of cluster RCTs in primary care showed that approximately two-thirds have clusters of unequal size [19]. Methods have already been proposed for inflating sample size to take into account such variability, the simplest of which rely on knowledge of the range of cluster sizes to be included [12]; however, many assume an average cluster size and do not take this into account when calculating sample size [20]. On the basis of the work presented herein, it might be expected that the impact of clusters merging may be less when the variability in cluster size has already been considered, but further work is needed to understand the consequences in this situation.

Although we have used primary care as the motivating example throughout this article, given the reduction over time in the number of GP practices within the United Kingdom [3], the results presented herein can be applied to other areas if there is a risk of cluster merges.

We have not yet considered other ways in which the cluster composition may change, such as merges with clusters not originally participating in the RCT, which is not likely to lead to biased estimates, but power is likely to be affected as the cluster size increases or if more than two clusters are merged. In addition, clusters may fragment, resulting in more clusters of smaller average size. Again, treatment estimates will be unbiased if original treatment allocation applies, but power will be affected. However, consideration would need to be given to whether these ‘new’ clusters should remain in the same treatment arm of the RCT, because it might be more appropriate to randomise if cluster members are to participate. Cluster membership may also fluctuate during the course of the study without merging or fragmentation of clusters, particularly in primary care, where patients leave and join a practice, an issue discussed by Diehr *et al.*[21] in relation to survey design.

The CONSORT extension for cluster RCTs requires the flow of clusters, as well as the flow of patients, to be described. Our review of the literature indicates that, even when authors have revealed changes to clusters, they did not do so in a manner that allowed full understanding. Clearly, authors need to follow reporting guidelines more closely, and journal editors should emphasise the need to do so. Investigators also need to consider whether changes need to be made to protocols, either to preempt any possible changes to cluster composition, defining up front how they should be dealt with or in response to such changes.

## Conclusions

Adjusting the design effect in power calculations for variability in cluster size and changes in average cluster size, we note that merging of clusters in cluster RCTs is expected to result in a loss of power. However, the simulations conducted examining homogeneous cluster merges resulted in a much smaller loss of power, to the extent of being largely unimportant, because the observed ICC decreased. This suggests that the relationship of ICC with cluster size should not be ignored at the planning stage.

A pragmatic approach in which the merged clusters are analysed as one new cluster, following homogeneous cluster merges, results in acceptable treatment effect estimates, so such merges should not cause concern. However, heterogeneous merges are problematic, leading to biased treatment effect estimates unless merged clusters are discontinued. If such clusters are discontinued, the estimate is unbiased, but with a loss of precision. Allowance for loss to follow-up at the cluster level as well as at the individual level might be advisable at the planning stage of a cluster RCT. Further research is warranted to fully understand the impact of other changes to clusters postrandomisation and to develop appropriate approaches to statistical analysis.

## Abbreviations

CONSORT: Consolidated Standards of Reporting Trials; GP: General practitioner; ICC: Intracluster correlation coefficient; RCT: Randomised controlled trial.

## Competing interests

The authors declare that they have no competing interests.

## Authors’ contributions

KLS conceived and designed the study and wrote the draft manuscript. NC participated in the study design, reviewed reports of trials, developed a program to explore impact on study power and contributed to the simulation study. MJGB participated in the study design, reviewed reports of trials and contributed to the simulation study. LG reviewed reports of trials and surveyed trial authors and clinical trial units. All authors commented on drafts of the manuscript and read and approved the final manuscript.

## Supplementary Material

Additional file 1 **Simulation code.** Example code (in Stata 12 software) for the simulations conducted to explore the impact of cluster merging. Click here for file
