# Supplementary material for: Changing cluster composition in cluster randomised controlled trials: design and analysis considerations
Source: Trials. 2014 May 24;15:184. doi: 10.1186/1745-6215-15-184 (PMC4039551; doi:10.1186/1745-6215-15-184)
Supplement: Additional file 1 — Simulation code. Example code (in Stata 12 software) for the simulations conducted to explore the impact of cluster merging. [file 1745-6215-15-184-S1.docx]

*********************************************

*** Simulating a CRT dataset ***

*********************************************

clear all

**/* Using the model Y_{ijk} = beta_{0k} + beta_1 + u_{0j} + e_{ij}**

**where**

**Y is the outcome for individual i in cluster j**

**beta_{0} is the mean outcome in treatment group 1 (the intercept)**

**beta_{1} is the treatment effect (i.e. the difference between treatment group means, mu2-mu1)**

**u_{0j} is the random effect in cluster j, u_{0j} ~ N(0, sigma_{b})**

**e_{ij} is the random effect at individual level, e_{ij} ~ N(0, sigma_{w})**

**Allow the user to specify true parameter values, as well as:**

**1. Number of clusters in each arm (C1 and C2)**

**2. Average cluster size**

**3. Number of homogeneous and heterogeneous merges**

**4. How to allocate (non-completing) individuals after a heterogeneous merge**

**5. Proportion of individuals who are “completers” at the point of the cluster merge**

***/**

capture program drop crtdatasim

program define crtdatasim

syntax, M(int) C1(int) C2(int) MU1(real) MU2(real) SIGMAW2(real) SIGMAB2(real) ///

[mergehom mergehet hommerges1(int 0) hommerges2(int 0) hetmerges(int 0) ///

hetgroup(int -1) COMPLETERs COMPpr(real 1) dropmerged]

clear

di "Note: this simulation assumes the following:" _newline ///

"1. There are 2 treatment groups" _newline ///

"2. All clusters are of equal size" _newline ///

"3. Within cluster variance is constant across clusters" _newline ///

"4. Between cluster variance is constant across treatment groups" _newline

local sigmaw = sqrt(`sigmaw2')

local sigmab = sqrt(`sigmab2')

local c=`c1'+`c2'

local obs = `m'*`c'

qui set obs `obs'

**/* Set up patient and cluster ids */**

egen i=seq(), from(1) to(`obs')

label variable i "Patient ID"

egen j=seq(), from(1) to (`c') block(`m')

label variable j "Cluster ID"

**/* Set up the individual errors */**

drawnorm inderr, means(0) sds(`sigmaw')

**/* Set up the cluster-level errors (using the norvec sub-program, which is defined below) */**

norvec `c' 0 `sigmab'

qui gen cluserr=.

forvalues k=1/`c' {

qui replace cluserr=`r(u0`k')' if j==`k'

}

**/* Set up treatment groups */**

local mc1=`m'*`c1'

egen trt=seq(), from(1) to(2) block(`mc1')

label variable trt "Treatment group"

**/* Set up within cluster patient ids */**

egen within_i=seq(), from(1) to(`m')

**/* Set up indicator for COMPLETERS */**

local m_C = ceil(`m'*`comppr')

gen completer = within_i<=`m_C'

label variable completer "Completed intervention before merge(s)"

**/* Set up the outcome variable */**

qui gen y=.

label variable y "Outcome"

forvalues k=1/2 {

qui replace y=`mu`k''+inderr+cluserr if trt==`k'

}

**/* Perform merges */**

local start1=1

local start2=`c1'+1

local end1 = `c1'

local end2 = `c'

**/* Homogeneous */**

if "`mergehom'"~="" {

gen merged=0

forvalues trtgroup=1/2 {

forvalues i=1/`hommerges`trtgroup'' {

local clusterno1=`start`trtgroup''

local MERGE1IND=0

while `MERGE1IND'==0 {

count if j==`clusterno1' & trt==`trtgroup' & merged==0

if `r(N)'>0 | `clusterno1'>`end`trtgroup'' {

local MERGE1IND=1

}

else local ++clusterno1

}

local clusterno2=`clusterno1'+1

local MERGE2IND=0

while `MERGE2IND'==0 {

count if j==`clusterno2' & trt==`trtgroup' & merged==0

if `clusterno2'>`end`trtgroup'' local MERGE2IND=1

else {

if `r(N)'>0 {

replace j=`clusterno1' if j==`clusterno2'

local MERGE2IND=1

}

else local ++clusterno2

}

}

replace merged=1 if j==`clusterno1'

}

}

}

**/* Heterogenous */**

if "`mergehet'"~="" {

capture gen merged=0

gen hetmerged=0

**/* Adjust outcome for non-completers (minus the trt group mean and**

**replace it later with the trt group mean after merging) */**

forvalues k=1/2 {

qui replace y=y-`mu`k'' if trt==`k' & completer==0

}

forvalues i=1/`hetmerges' {

local clusterno1=`start1'

local MERGE1IND=0

while `MERGE1IND'==0 {

count if j==`clusterno1' & trt==1 & merged==0

if `r(N)'>0 | `clusterno1'>`end1' {

local MERGE1IND=1

}

else local ++clusterno1

}

local clusterno2=`start2'

local MERGE2IND=0

while `MERGE2IND'==0 {

count if j==`clusterno2' & trt==2 & merged==0

if `clusterno2'>`end2' local MERGE2IND=1

else {

if `r(N)'>0 {

replace j=`clusterno1' if j==`clusterno2'

local MERGE2IND=1

}

else local ++clusterno2

}

}

replace merged=1 if j==`clusterno1'

replace hetmerged=1 if j==`clusterno1'

}

if `hetgroup'==1 | `hetgroup'==2 {

replace trt=`hetgroup' if hetmerged==1

}

else drop if hetmerged==1

if "`completers'"=="completers" {

drop if completer==0 & hetmerged==1

}

**/* Adjust outcome for non-completers (add the trt group mean) */**

forvalues k=1/2 {

qui replace y=y+`mu`k'' if trt==`k' & completer==0

noi di "note: mu_`k' = `mu`k''

}

}

**/* Drop merged clusters if specified */**

if "`dropmerged'"~="" drop if merged==1

save crtdata, replace

end

****************************

*** norvec (sub) program ***

****************************

capture program drop norvec

program define norvec, rclass

args n mu sigma

preserve

clear

qui set obs `n'

drawnorm x, means(`mu') sds(`sigma')

mkmat x, mat(u0)

forvalues k=1/`n' {

return scalar u0`k'=u0[`k',1]

}

end

/* args m c1 c2 mu1 mu2 sigmaw2 sigmab2 */
